# Building of a composite virtual slide from contiguous tissue samples

**DOI:** 10.1186/1746-1596-9-S1-S9

**Published:** 2014-12-19

**Authors:** Benoît Plancoulaine, Myriam Oger, Nicolas Elie, Philippe Belhomme, Paulette Herlin, Abir Nasri, Célia Augé, Mylène Brécin, Jacques Marnay, Catherine Bor-Angelier

**Affiliations:** 1 Normandie Université; UNICAEN, CLCC F. Baclesse, PATHIMAGE BioTICLA EA 4656, Caen, France; 2 CLCC F. Baclesse, Pathology department, Caen, France; 3 Normandie Université; UNICAEN, CMABIO-HIQ facility, Caen, France

## Abstract

**Background:**

Currently available microscope slide scanners produce whole slide images at various resolutions from histological sections. Nevertheless, acquisition area and so visualization of large tissue samples are limited by the standardized size of glass slides, used daily in pathology departments. The proposed solution has been developed to build composite virtual slides from images of large tumor fragments.

**Materials and methods:**

Images of HES or immunostained histological sections of carefully labeled fragments from a representative slice of breast carcinoma were acquired with a digital slide scanner at a magnification of 20×. The tiling program involves three steps: the straightening of tissue fragment images using polynomial interpolation method, and the building and assembling of strips of contiguous tissue sample whole slide images in × and y directions. The final image is saved in a pyramidal BigTiff file format. The program has been tested on several tumor slices. A correlation quality control has been done on five images artificially cut.

**Results:**

Sixty tumor slices from twenty surgical specimens, cut into two to twenty six pieces, were reconstructed. A median of 98.71% is obtained by computing the correlation coefficients between native and reconstructed images for quality control.

**Conclusions:**

The proposed method is efficient and able to adapt itself to daily work conditions of classical pathology laboratories.

## Background

Microscopes associated with modern acquisition systems, such as video camera, give the pa-thologist opportunity to work, on screen, at different magnifications [1]; however it is still difficult to visualize, in one time, the entire histological preparation. Since less than two decades [2], microscope slide scanner devices give access to an image of the whole histological section [3], so-called "Whole Slide Image" (WSI). It allows a quick inspection of the tissue fragment by panning into its entire section surface and by zooming progressively across the continuous magnification scale [4], thanks to a virtual slide viewer. Nevertheless, despite this technological advance, the tissue surface that can be observed on the computer screen is classically limited to the dimensions of the standard glass slides and glass coverslips used daily in pathology laboratories. It is always possible to generate a WSI of large tissue fragments, using large slides and microscope slide scanners that accept them. However, the technical and economic constraints limit the use of this technology to research studies and specialized laboratories and can be hardly introduced in a routine pathology department. Recently published papers described a technique to rebuild a whole prostatic tumor section from WSI of four contiguous tissue fragments of standard size [5,6], using a mainly interactive method.

The aim of the present paper is to detail a solution able to give one high resolution large image of the whole histological section of big lesions, called Composite Virtual Slide (CVS), rebuilt from any number of WSI of contiguous tissue fragments. A quality control protocol was settled in addition, in order to evaluate the quality of the results more precisely than only visually.

## Materials and methods

### Tissue samples

The solution was elaborated from surgical specimens of invasive breast carcinoma, 20 primary tumors and 1 metastatic lymph node, collected since 2011, from patients followed at the François Baclesse Cancer Centre.

### Sample preparation

The most representative slice of each surgical specimen is selected during the macroscopic examination. The whole slice is cut into several pieces according to a regular grid of rectangles whose size should fit those of a standard disposable embedding cassette (31 mm × 25 mm × 5 mm). At this stage, each fragment is oriented and carefully labeled according to its localization recorded on a photographic map. After paraffin embedding, each fragment is trimmed into 3 to 5 µm thick section; each thin paraffin section is spread over the glass slide, while carefully avoiding its upturn, and then submitted to standard protocols.

### Staining

The histological sections were stained according to the Hematoxylin-Erythrosine-Saffron (HES), Periodic Acid Schiff (PAS) and/or immunostained for hormonal receptors (ER and PR), HER2, proliferation markers (Ki-67 and PHH3), vascular marker (CD31), using automatons and standard protocols of the pathology department.

### Image acquisition

Whole slide images of histological sections were digitized at 20× using the ScanScope CS^® ^microscope slide scanner from Aperio Technologies. They were recorded as tiled tiff images.

### Image processing for WSI-tiling

Taking into account the large size of the WSI, a two resolution procedure has been developed. A first composite image is built at a low resolution. The user is allowed, at this step, to correct the automatic stitching of the fragments if needed. Then, the final composite virtual slide is assembled at full resolution by reference to the parameters of the previous adjustment done at low resolution.

**Figure 1:**
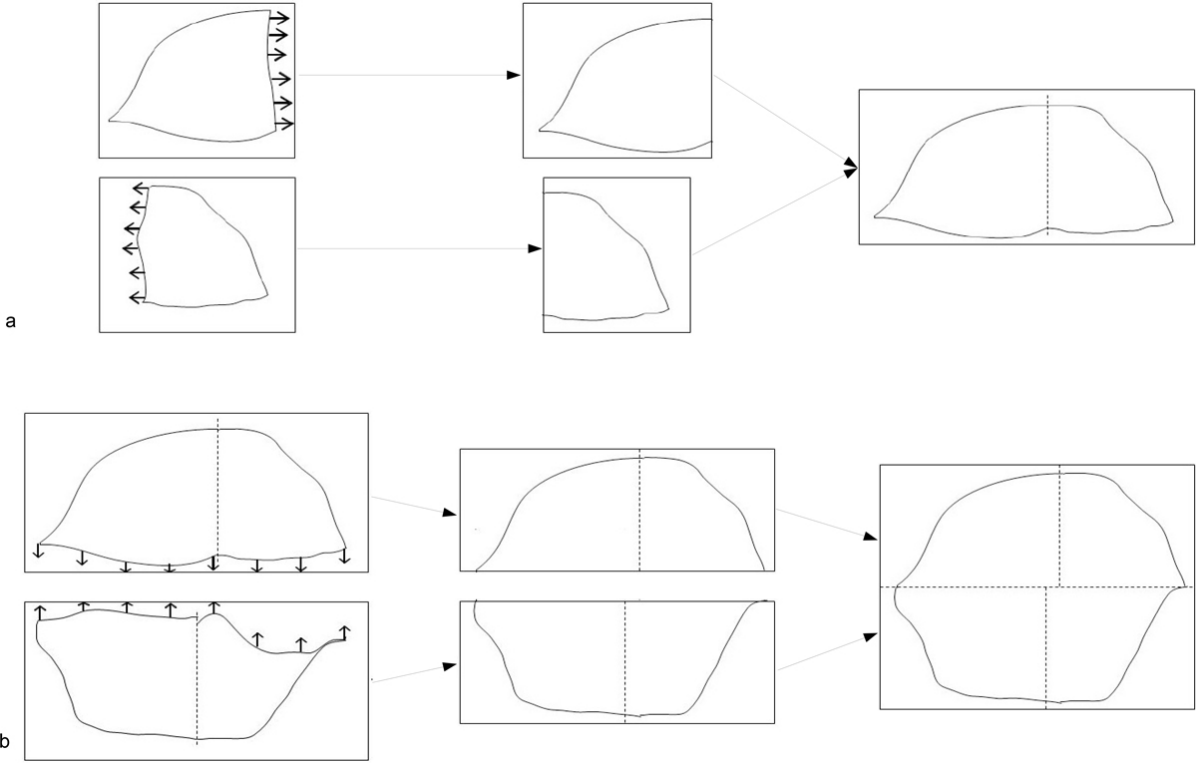
**Straightening and stitching of the binary masks**. Straightening and stitching of the binary masks of a) two tissue fragments to obtain an horizontal strip, b) two horizontal strips to obtain the composite image.

First, the program extracts a low resolution sub-image (8 µm per pixel) from the WSI pyramid of each fragment; it corresponds to a sixteen fold (x and y) down-sampling of the full resolution image (0.5 µm per pixel). Then it constructs the corresponding untiled binary mask of the tissue and computes its edges. The fully automatic program builds thereafter horizontal strips by bringing together recursively binary images of tissue fragments from the left to the right according to the original map. For each strip, it straightens first the fragment on the left to align its right-side edge pixels; it then straightens the fragment on the right to align its left-side edge pixels. The two fragments are thereafter registered by matching the right-side edge center of the first straightened fragment with the left-side edge center of the second straightened fragment of the tissue section (Figure 1a). Finally, the low resolution composite binary mask is built by assembling recursively downwards the horizontal strips of fragments previously stitched: the application straightens the top strip to align its bottom-part pixels, straightens the bottom strip to align its top-part pixels, then registers both strips by matching bottom-side center of the straightened top strip with the up-side center of the straightened bottom strip (Figure 1b).

A low resolution untiled color image is built in parallel taking the straightened binary masks as a reference, in order to visually evaluate the reconstruction result. At this step, if needed, the user can correct the original binary masks as well as the stitched strips with an image editing software in order to reduce artifacts at the edges. Since the algorithm uses the binary masks including any adjustments, the user can repeat this low resolution correction until a satisfactory reconstruction is obtained.

For the construction of the color high resolution composite virtual slide, each WSI and each strip are straightened and stitched by reference to the distortion parameters of the low resolution binary masks, refined by a polynomial interpolation. The final CVS is provided as a tiled "BigTiff" image.

The program was developed in C language without any parallelization and ran on 32 and 64 bits personal computers under Windows and Linux operating systems.

### Quality control protocol

This protocol consists of four steps which were applied on five breast tumors to evaluate the internal deformation of tissue structures. These tumors were chosen in order to get a representative whole section on a single glass slide (HES staining). First, the WSI was split in several pieces (4 to 17). Then, the entire section was reconstructed thanks to WSI-tiling program. Finally, each obtained CVS was compared to the original WSI. Due to the huge size of the images the comparison was done on a representative sample of them. For this purpose, a random systematic sampling (spacing of 2500 pixels) was done on the paired images, WSI and corre-sponding CVS. This sampling allows the extraction of a high resolution gallery of representative thumbnails of 500 × 500 pixels for each image. Each pair of thumbnails was registered [7] and the correlation coefficient was computed between these registered thumbnails. This procedure was reiterated four times.

Another control was done on images with less than 6 pieces to evaluate the influence of dis-tortions by rotation and homothety of each piece.

## Results

**Figure 2:**
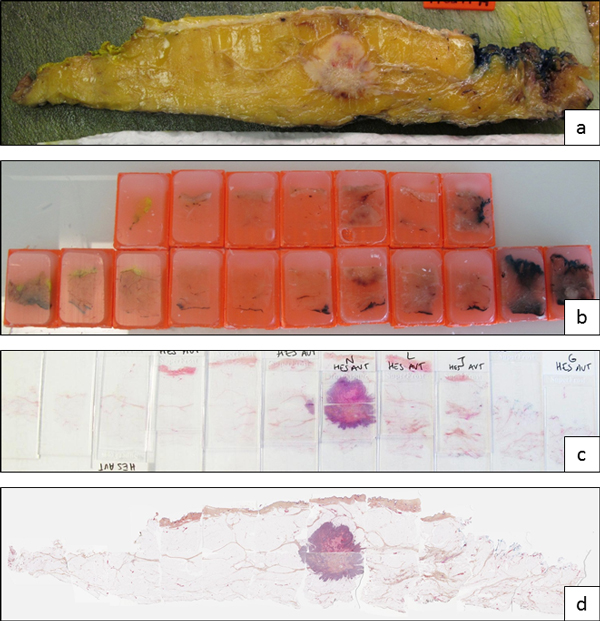
**From surgical specimen to Composite Virtual Slide**. a) Most representative slice of the surgical specimen; b) embedding cassettes containing all pieces of the entire slice; c) 5µm histological sections stained according to HES protocol; d) Composite Virtual Slide obtained after using the WSI-tiling strategy.

The full strategy used for getting Composite Virtual Slides is illustrated Figure 2.

Sixty images of a whole histological section of twenty slices of breast tumors were built from 2 to 26 WSI (table 1). Few seconds for 2 WSI, to five minutes for 26 WSI, were necessary to obtain a low resolution untiled color image. The automatic high resolution procedure, working on only one processor, has taken 6 hours for a CVS built from 10 WSI (patient #2, table 1) using a PC running under 64 bits operating system, with 2.4 GHz of i7 CPU and 16 Gb of RAM.

**Table 1:** Details of the 60 reconstructed images, ranked according to the size of the tissue slice.

	# Patient	Staining	# of WSI	Size in pixels	Size in GB	Size of the slice (mm)
1	1	HES	25	246576 × 425856	293.4	230 × 110
			
2		HES	26	246576 × 556080	383.1	

3	2	Ki67	10	101040 × 373024	105.3	200 × 55

4	3	HES	18	104384 × 513664	149.8	200 × 40
			
5		HES	6	104400 × 142592	41.6	
			
6		ER	6	104048 × 155248	45.1	
			
7		PR	6	104400 × 143392	41.8	
			
8		Ki67	6	98992 × 139424	38.6	
			
9		HES	6	104400 × 142592	41.6	

10	4	HES	8	250272 × 42816	29.94	200 × 30
			
11		HES	8	285376 × 83792	66.8	

12	5	HES	14	99680 × 449120	125.1	170 × 40

13	6	HES	17	332976 × 144848	134.8	150 × 80

14	7	HES	11	145792 × 286464	116.7	140 × 70
			
15		ER	11	143984 × 298256	120	
			
16		PR	11	145792 × 298992	121.8	
			
17		Ki67	11	147584 × 311872	128.6	
			
18		HER	11	143984 × 298256	120	
			
19		HES	7	151184 × 122064	51.6	
			
20		HES	17	161667 × 381488	172.3	

21	8a (Tumor)	ER	9	149392 × 193264	80.7	85 × 65
			
22		PR	7	147584 × 145888	60.2	
			
23		Ki67	7	150192 × 148928	62.5	

24	8b (Metastasis)	HES	4	109792 × 96384	29.6	45 × 50
			
25		PR	4	109792 × 93248	28.6	

26	9	HES	6	194384 × 88800	48.2	82 × 35

27	10	HES	3	163792 × 51280	23.5	80 × 25

28	11	HES	3	62432 × 141296	24.6	70 × 30

29	12	HES	3	134384 × 57680	21.7	65 × 35
			
30		ER	3	58896 × 122928	20.2	
			
31		PR	3	55456 × 128640	19.9	
			
32		Ki67	3	63600 × 127392	22.6	
			
33		HER	3	64528 × 122288	22	
			
34		HES	3	56256 × 135200	21.3	

35	13	HES	4	115200 × 82997	26.7	60 × 40
			
36		HES	4	116752 × 88896	29	
			
37		ER	4	109800 × 90343	27.7	
			
38		PR	4	109800 × 91497	28.1	
			
39		Ki67	4	115200 × 94360	30.4	
			
40		PAS	4	117000 × 86126	28.2	
			
41		PHH3	4	120600 × 90403	30.5	
			
42		CD31	4	117000 × 93036	30.4	

43	14	HES	4	118784 × 90032	29.9	55 × 35
			
44		ER	4	122400 × 88240	30.2	
			
45		PR	4	122384 × 89216	30.5	
			
46		Ki67	4	116992 × 88928	29.1	

47	15	HES	2	100800 × 48016	13.5	55 × 25
			
48		ER	2	98992 × 49584	13.7	
			
49		PR	2	95392 × 49744	13.3	
			
50		HES	2	100784 × 48336	13.6	

51	16	HES	2	72000 × 82544	16.6	50 × 35
			
52		ER	2	61120 × 79440	13.6	
			
53		PR	2	61200 × 81744	14	
			
54		Ki67	2	62992 × 80336	14.1	
			
55		HES	2	73184 × 85488	17.5	

56	17	HES	2	52928 × 89344	13.2	50 × 25
			
57		HES	2	52192 × 87456	12.8	

58	18	HES	2	91792 × 40688	10.4	50 × 20

59	19	HES	2	59808 × 88256	14.7	45 × 25

60	20	HES	2	66816 × 83872	15.7	40 × 30

**Figure 3:**
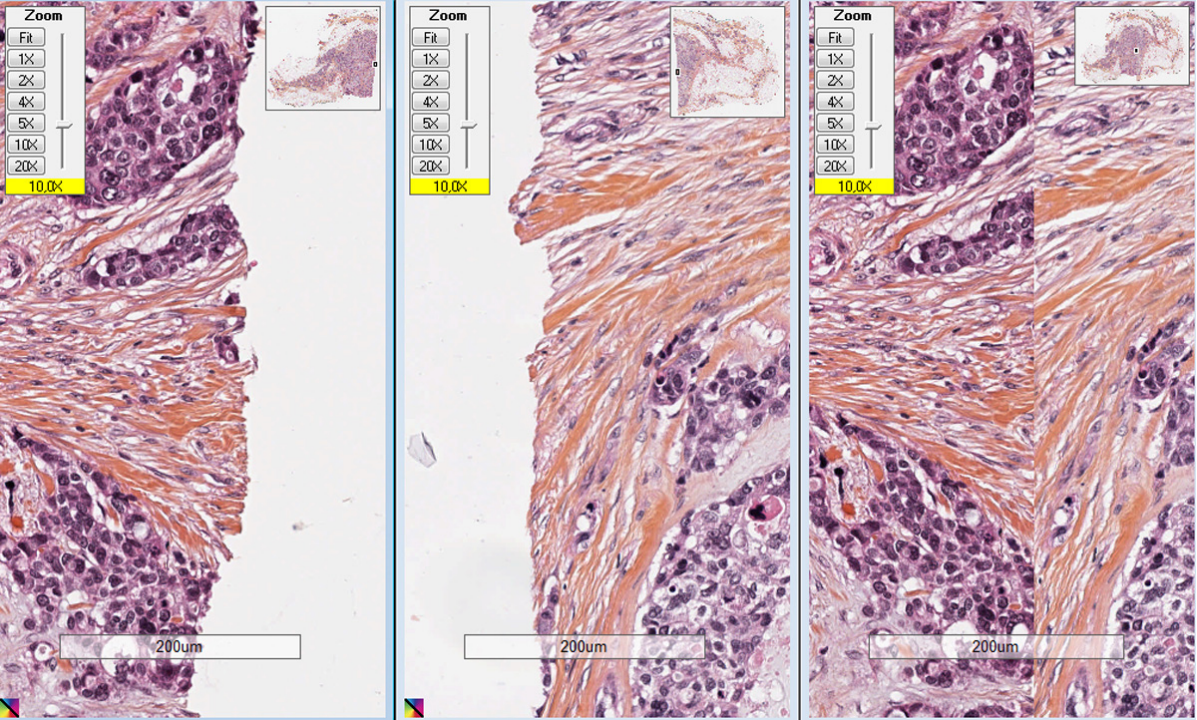
**Detail of a stitching zone after WSI-tiling strategy**. Left: detail of the left WSI; center: detail of the right WSI; right: final CVS coming from two WSI.

Figure 3 illustrates the visual quality of the straightening of boundaries and internal tissue of WSI inside the final CVS. During the stitching process, when the limits of the tissue fragments are uneven, some small parts of the limits of the tissue can be lost.

**Figure 4:**
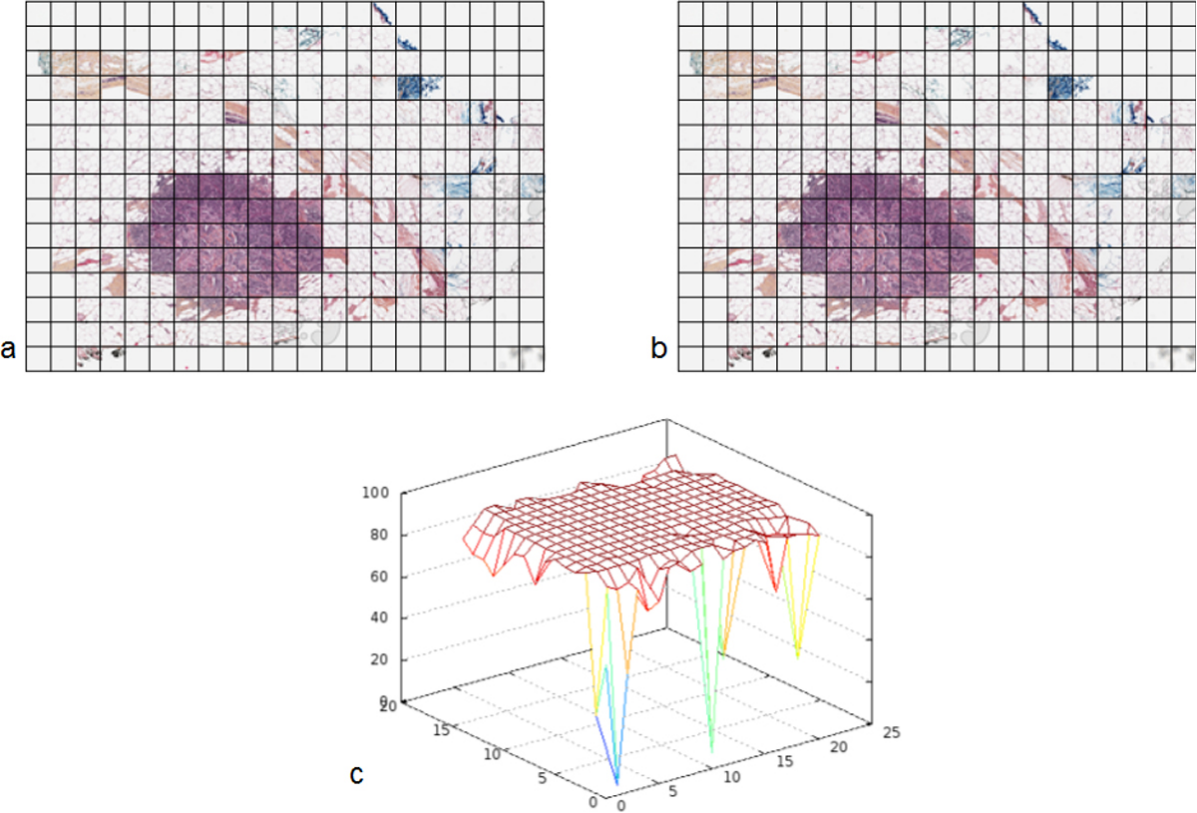
**Example of quality control result**. a) Gallery of the sampled original WSI; b) Gallery of the sampled CVS; c) 3D representation of the correlation coefficient between pairs of registered thumbnails.

Results of the quality control are presented Figure 4 and table 2. Figure 4 illustrates, for one of the tested cases and one random systematic sampling, the correlation obtained, thumbnail per thumbnail, between galleries of the original image and the corresponding CVS. In this example, the majority of thumbnails exhibits a correlation coefficient close to 100%. The worse correlation coefficients are obtained from background white thumbnails, for which no registration could be done. The table 2 gives the median of the correlation coefficients of thumbnails coming from the CVS rebuilt from non-distorted WSI. The median of the correlation coefficients of the five cases for non-distorted WSI is 98.71%. After rotation or homothety this median falls respectively to 87.57% (n = 2) and 85.90% (n = 2) and reaches 80.73% when combining the two (n = 3).

**Table 2:** Quality control results: Correlation coefficients computed on pairs of high resolution thumbnail galleries from CVS.

Image	number of fragments per image	Sampling	number of thumbnails per gallery	Correlation coefficient
Image 1	5	1^st^	90	98.70%
		
		2^nd^	90	98.51%
		
		3^rd^	90	98.45%
		
		4^th^	90	98.44%

Image 2	4	1^st^	20	98.14%
		
		2^nd^	16	97.26%
		
		3^rd^	16	97.40%
		
		4^th^	16	98.46%

Image 3	17	1^st^	315	99.23%
		
		2^nd^	330	99.25%
		
		3^rd^	315	99.23%
		
		4^th^	315	99.22%

Image 4	12	1^st^	396	98.70%
		
		2^nd^	418	98.64%
		
		3^rd^	396	98.72%
		
		4^th^	396	98.84%

Image 5	6	1^st^	238	99.25%
		
		2^nd^	208	99.24%
		
		3^rd^	221	99.27%
		
		4^th^	221	99.23%

## Discussion

The proposed solution has proved its efficacy to rebuild composite high resolution virtual slides of very large breast tumor slices cut into several tissue fragments included in classical embedding cassettes.

The reconstruction of the high resolution CVS is fully automatic, but at low resolution, a user intervention is possible to improve the stitching, if necessary. The WSI-tiling procedure is ready for use and accepts classical Tiff as well "BigTiff" tiled images.

The high resolution reconstruction is long, but the decrease of the process time is to be con-sidered by optimizing the code with the introduction of parallelization in order to take advantage of the multi-cores available on classical PC.

The chosen quality control strategy showed satisfactory quantitative performances. Even if the performances of the proposed solution lightly decrease when the fragments are previously distorted, the correlation coefficient is still high, and the quality of the final image is almost identical to the initial image. This decrease can come from both the artificial distortion (interpolation effect) and the straightening used by the here proposed solution.

To limit the distortions inside the final CVS, coming from initial deformations of tissue fragments, it is then mandatory to carefully orientate tissue fragments in the embedding cassettes as well as paraffin ribbons on glass slides.

Even if some scanner devices can accept large slides, the benefit of the proposed solution is its ability to adapt to the daily work conditions and economic constraints of common pathology departments.

To the best of our knowledge, two papers of the same team have already described a tech-nique to rebuild the whole prostatic tumor section from WSI of contiguous tissue fragments [5,6]. But, the proposed method is interactive, its use is limited to four fragments and up to now, it did not give access to the reconstruction of an image at a magnification higher than 10×. In this context, the here proposed solution has been developed to give the pathologist the ability to observe and make measurements, up to the resolution of 0.5 µm (i.e. magnification of 20×), on one large image of the whole histological section of large lesions, rebuilt from any number of virtual slides of contiguous tissue fragments, while limiting at most the user inter-vention.

Specific viewer allows the "stitching" of contiguous slides without rebuilding the CVS. Nev-ertheless, to process and analyze the whole tumor slice, a rebuild CVS is necessary.

The added values of this solution are numerous for the pathologist, contributing both in rou-tine diagnosis and in research.

This solution would contribute to make an easier histological diagnosis on surgical specimen. The reconstruction of an entire section of an organ on virtual slides could help pathologist to finely evaluate the surgical margins, the invasion and growth of the lesion as well as the abnormalities of the adjacent structures morphology [8], on a faster and easier way. So this solution might potentiate the interest of using digital slides routinely.

In research, this approach could be of great interest both for correlative research with other image modalities [8,9] (macro-images of the tumor section, scanner, MRI or ultra-sound images) and to better understand these images in translational research. Image analysis could help studying architectural features and heterogeneity of distribution of immunohistochemical markers on large tumors, and select cell immunophenotypic clones for genetic analysis. The registration of serial histological sections and study of co-localization of markers could be possible. So this solution might contribute to the understanding of different ways of tumor growth.

## Conclusions

This solution will have further developments in order to create an interface for users and to improve speed for daily practice. As it could of course contribute to research giving topographic information on tissues, the use of such a solution is one of the first steps expected by the pathologists before adopting digital pathology for their routine diagnosis. The ability to visualize complete sections of any surgical specimen could facilitate pathologists' daily work, giving them an easy access to information, such as margins and size of tumor structures, which are time consuming steps. Even more, the use of composite virtual slide will give more accuracy to measurements; the precision of these parameters is required by oncologists for further therapeutic decisions.

## Competing interests

The authors declare that they have no competing interests.

## Authors' contributions

BP: Developed study conception and design, and drafted the manuscript. MO: Selected cases, performed data acquisition, performed image processing and quality control and drafted the manuscript. NE, PB, HP, JM: participated in the design of the study and drafted the manuscript. AN, CA, MB: Developed and performed sample preparation protocol, selected cases and performed sample preparation, slide staining and data acquisition. CB: Supervised study concept and design, selected cases, performed histopathological evaluation and drafted the manuscript. All authors read and approved the final manuscript.
